# Pericardial Cyst: An Uncommon Cause of Fever

**DOI:** 10.7759/cureus.74900

**Published:** 2024-12-01

**Authors:** Rodrigo Duarte, Raquel Flores, João Pereira

**Affiliations:** 1 Internal Medicine, Centro Hospitalar Lisboa Ocidental, Lisbon, PRT; 2 Internal Medicine, Hospital Egas Moniz, Lisbon, PRT

**Keywords:** fever of unknown origin (fuo), pericardial cysts, pericardial masses, symptomatic pericardial cyst, syndrome of fever of unknown origin

## Abstract

Pericardial cysts are a rare and benign entity that comprise 7% of the mediastinal masses. They are asymptomatic in over half of the cases, being usually detected as an incidental mass lesion on chest X-ray. When symptomatic, they usually present with dyspnea, chest pain, or persistent cough. Fever at presentation is an uncommon symptom. We present the case of a 40-year-old woman with a history of fever of unknown origin for over two years. The diagnostic study was relevant for an echocardiogram showing a pericardial cyst in the right cardiophrenic angle. While waiting for surgical resection, the patient developed heart failure refractory to diuretic therapy. Surgical resection of the cyst resulted in the resolution of her complaints with no further episodes of fever.

## Introduction

Pericardial cysts are a rare and benign entity that comprise 7% of the mediastinal masses [1]. Its incidence is one in 100,000 population, affecting males and females equally, and is usually found in the third or fourth decade of life [1]. These cysts are located in the right cardiophrenic angle in about 70% of the cases, followed by the left costophrenic angle, hilum, and superior mediastinum [2,3]. Pericardial cysts are often asymptomatic (50% to 70%) and are usually diagnosed incidentally using radiological investigations such as chest X-rays or CT [4-6]. When symptomatic, they present with dyspnea, chest pain, or persistent cough. Unusual presentations include recurrent syncope, fever, pneumonia, congestive heart failure, and sudden cardiac death [3,4]. We present the case of a female patient with a prolonged fever in which the only relevant diagnostic finding was a pericardial cyst.

## Case presentation

A 40-year-old female patient presented to the internal medicine department due to a fever over the last six months. It had a maximum axillary temperature of 38.5ºC with irregular periodicity that was alleviated with antipyretics. It was accompanied by nocturnal sudoresis, anorexia, asthenia, and weight loss of 7 kg in the last six months. She referred to sporadic retrosternal pain without irradiation, nor relation with physical activity or swallowing, of variable intensity and duration. She referred to dyspnea for moderate exercise without orthopnea, nocturnal paroxysmal dyspnea, peripheral edema, gastrointestinal or urinary symptoms, or skin involvement. She had already undergone a cycle of intravenous antibiotic therapy (which she could not specify) for three days at the beginning of the whole condition, with no improvement.

The patient had no relevant medical history nor allergies and a familiar history of a pulmonary lymphoma in her father, a treated non-Hodgkin lymphoma in her daughter, esophageal cancer in her uncle, and ischemic cardiopathy in her grandparents. From the past medical history, it was found that there was contact with cattle, professional contact with a patient infected with tuberculosis, and ingestion of unpasteurized cheese. The patient denied recent travels, risky sexual contacts, or tick bites. The patient had two dogs, both vaccinated and dewormed.

Upon physical examination, the patient had a good general condition, was anxious, normotensive, and eupneic, with normally colored and hydrated skin and mucous membranes, without skin lesions. There was no jugular distension, thyroid nodules, or enlargement of the thyroid; it was unpainful to palpation. No palpable lymph nodes nor changes in cardiac, pulmonary, or abdominal examination were present. There was no peripheral edema.

**Figure 1:**
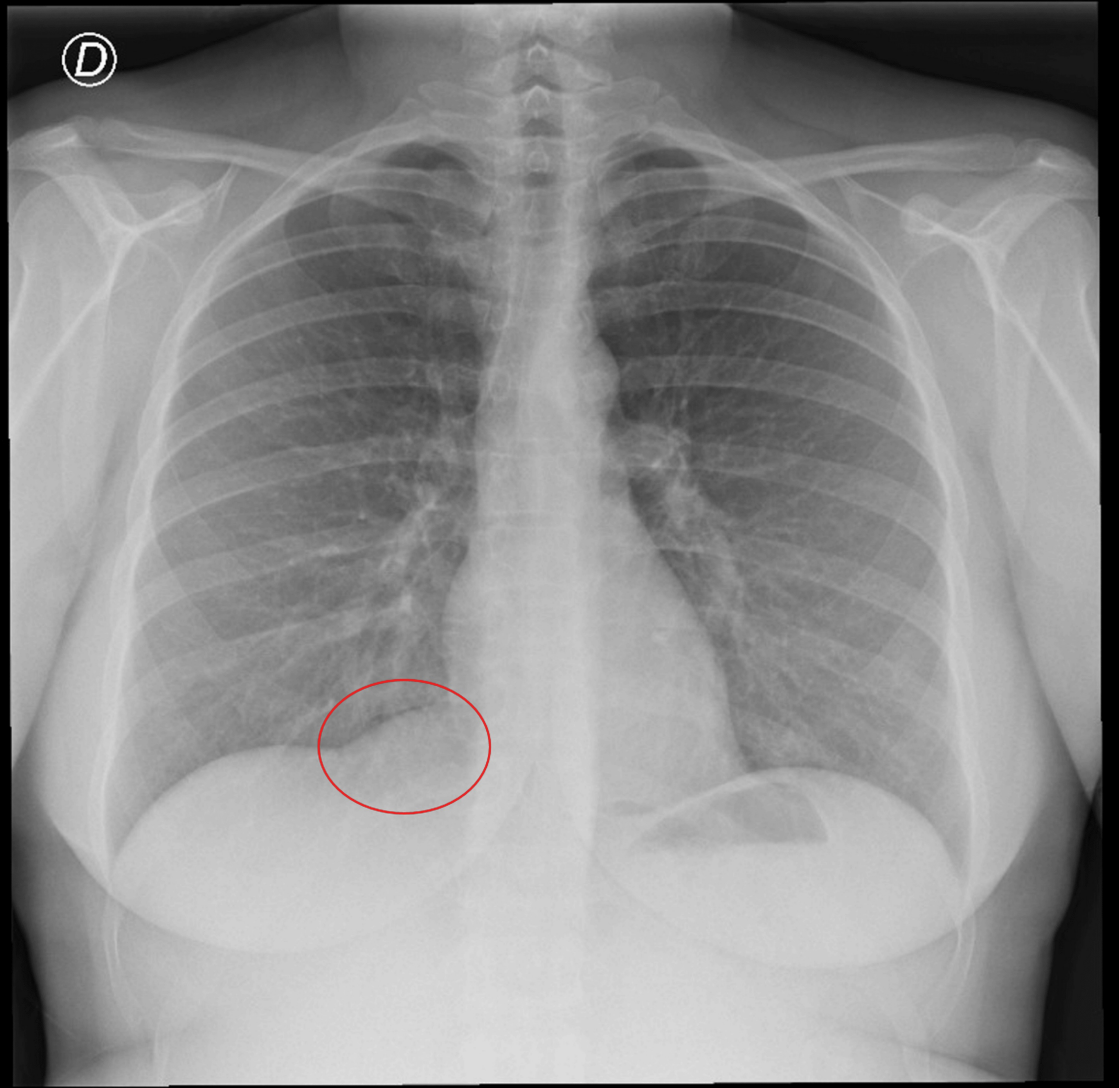
Chest X-ray of the patient shows a slight elevation of the right cardiodiophrenic angle (red circle)

A complete analytical study with protein electrophoresis, autoimmunity (specifically antinuclear antibodies (ANAs), antineutrophil cytoplasmic antibodies (ANCAs), anti-DNA), and serologies (HIV 1 and 2, hepatitis C virus (HCV), hepatitis B virus (HBV), cytomegalovirus (CMV), Epstein-Barr virus (EBV)), urine and blood cultures, Mantoux test, abdominal and thoracic CT scans, abdominal MRI, transthoracic echocardiogram, and bronchoscopy was requested. The chest X-ray is shown in Figure 1. Of the mentioned exams, only the echocardiogram showed the presence of a pericardial cyst (4.5 cm x 3 cm) at the right cardiophrenic angle. There were no changes in segmental contractility or compression of the right cavities, and the left ventricular ejection fraction was preserved. 

**Figure 2:**
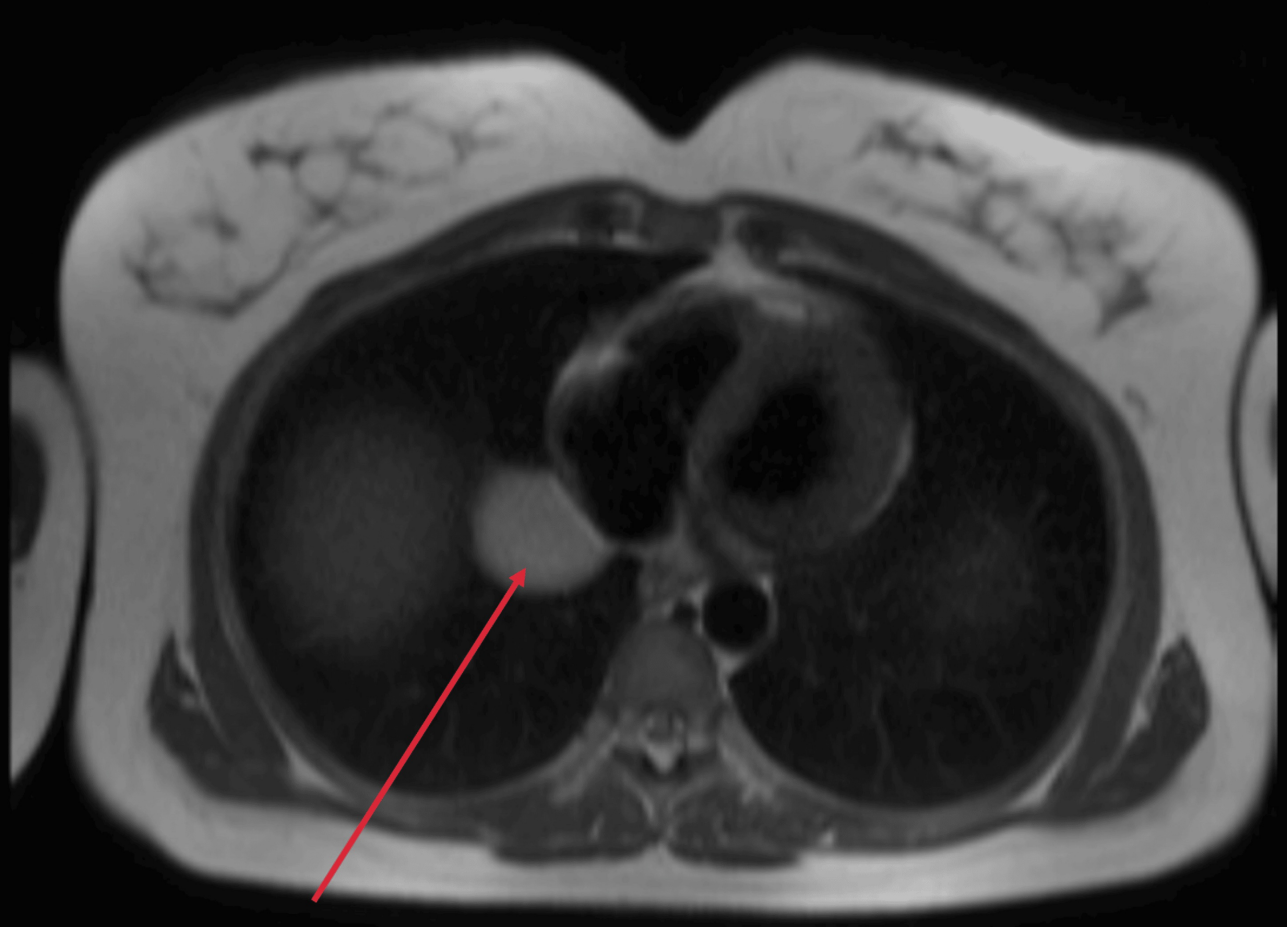
Transverse view of the cardiac MRI. The red arrow points to the pericardial cyst.

**Figure 3:**
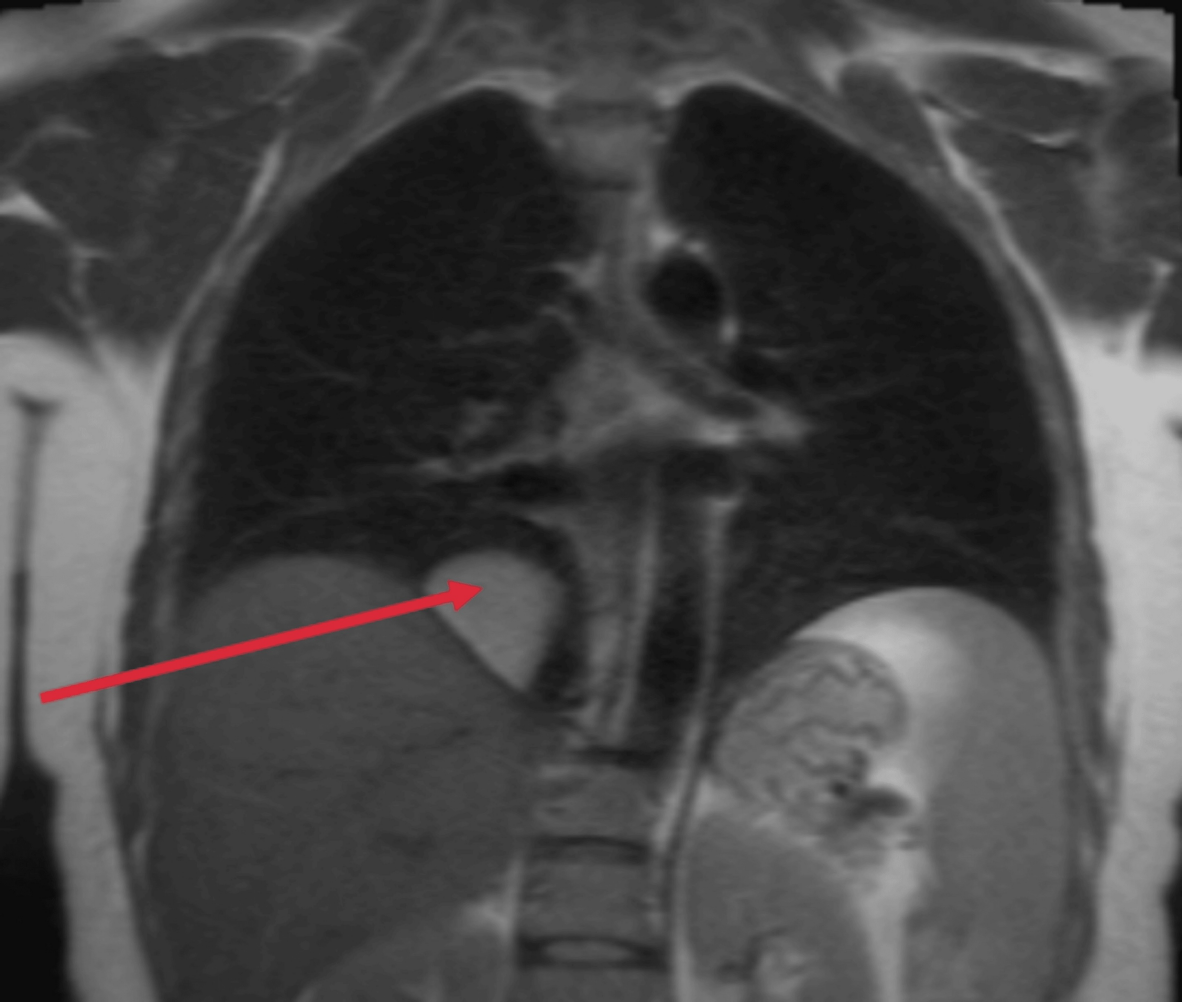
Coronal view of the cardiac MRI. The red arrow points to the pericardial cyst.

**Figure 4:**
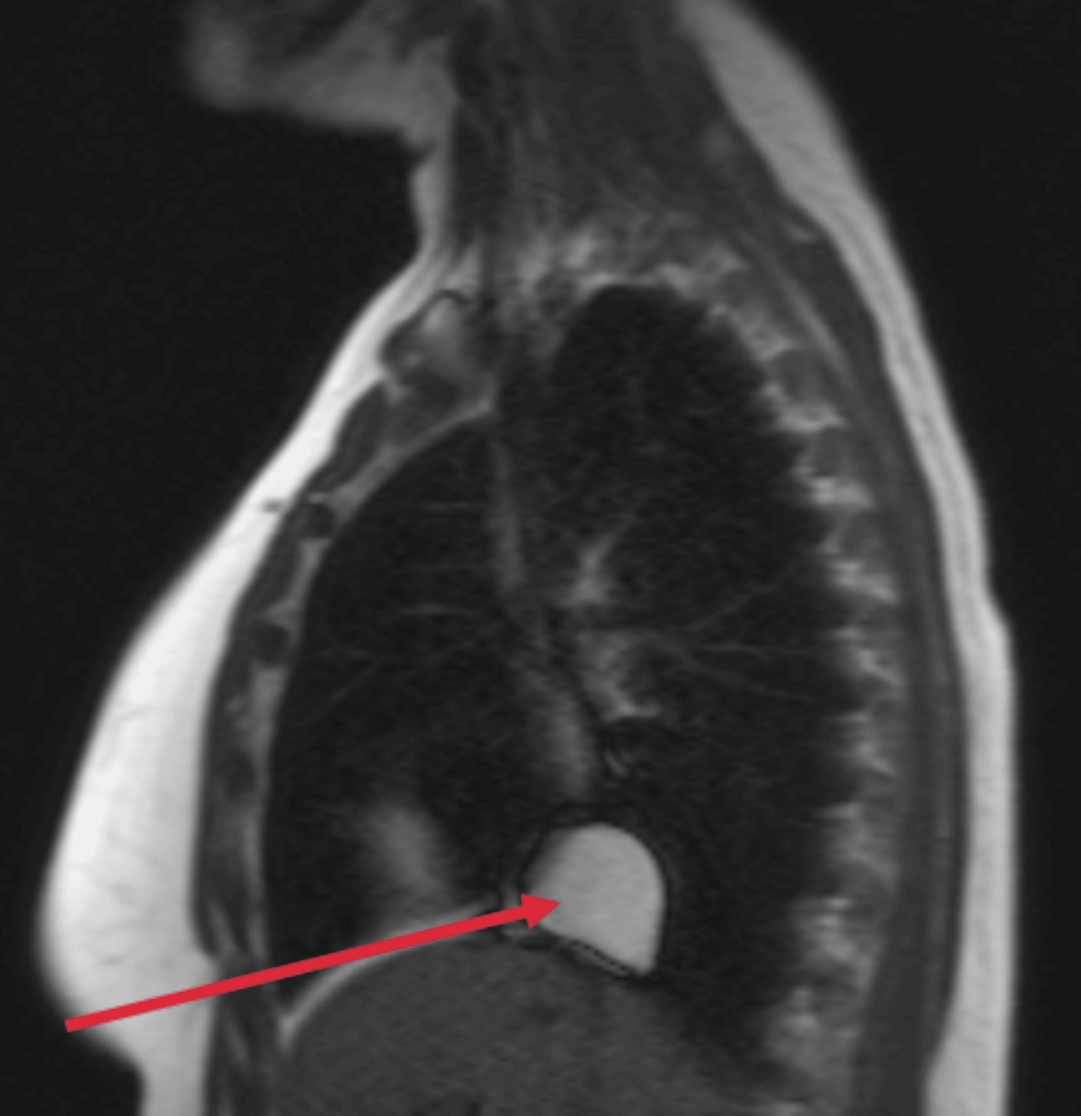
Sagital view of the cardiac MRI. The red arrow points to the pericardial cyst.

New serologies were requested, including interferon-gamma release assay (IGRA) testing for tuberculosis, *Brucella* spp. (Rose Bengal), *Mycoplasma* spp., *Chlamydia* spp., *Legionella* spp., *Treponema pallidum*, *Coxiella burnetii*, *Borrelia *spp., and *Echinococcus* spp.; all tested negative. A cardiac MRI (Figures 2-4) was performed to better characterize the cyst. The findings were compatible with a pure pericardial cyst, located in the retroauricular right region, without compressing adjacent structures. The presence of fibrosis was unable to be assessed due to the lack of late gadolinium-enhanced sequences directed at studying the mass. 

During the follow-up in subsequent consultations over two years, fever was never observed. However, the patient continued to report high temperatures with characteristics similar to those described (up to 38 ºC). Eventually, she started to develop symptoms of heart failure with the onset of fatigue to small efforts, episodes of orthopnea, and paroxysmal nocturnal dyspnea, even with diuretic therapy. The patient was then sent to the cardiothoracic team for surgical excision of the pericardial cyst, which was conducted without complications. The cyst was not sent for anatomopathological examination. Six months after the surgery, the patient reported no more fever and remission of her complaints. She was discharged from the internal medicine department.

## Discussion

A pericardial cyst is a rare benign congenital lesion of the mediastinum [7]. It is typically an asymptomatic entity, incidentally found during radiological exams [1,4,6]. Fever is not a common clinical manifestation of the pericardial cyst. A review of 114 cases by Najib et al. found only one instance of fever at presentation [3,8]. Additionally, Amr et al., in a literature review of pericardial cysts complicated with acute cardiac tamponade, noted fever in four of nine reviewed cases; this remains an uncommon finding [9]. 

The patient in this case presented with a fever of unknown origin for two years, without positive results for the etiology of her fever after an extensive workup. The only relevant finding was a pericardial cyst in the retro-auricular right region, with a cardiac MRI showing a pure cyst without compression of adjacent structures. The cyst was eventually complicated with heart failure and pulmonary edema, refractory to the medical therapy implemented. As there was no anatomopathological examination performed, due to the benign findings of a pure pericardial cyst in the imaging modalities, the authors interpreted it as an idiopathic cyst.

As there was complete remission of the fever and the heart failure symptoms with the cysts’ resection, the authors believe that those symptoms were due to the pericardial cyst. It should be noted that an untreated pericardial cyst may evolve and cause compression of nearby structures. Rarely, it can be associated with severe complications such as heart failure, cardiac tamponade, rupture, and, sometimes, sudden cardiac death.

## Conclusions

Pericardial cysts are rare entities and frequently underestimated. Patients are usually asymptomatic or have dyspnea, chest pain, or persistent cough as the most common symptoms. Large cysts might lead to serious complications such as cardiac tamponade and sudden death. Symptomatic cysts should be treated by aspiration or surgical resection. Despite being an uncommon manifestation, fever can occur, and the presence of progressive symptoms should prompt the physician for early intervention in order to prevent its potential complications.
